# Initiation and propagation of α-synuclein aggregation in the nervous system

**DOI:** 10.1186/s13024-020-00368-6

**Published:** 2020-03-06

**Authors:** Baraa A. Hijaz, Laura A. Volpicelli-Daley

**Affiliations:** Department of Neurology, Center for Neurodegeneration and Experimental Therapeutics, University of Alabama at Birmingham, Birmingham, AL 35294 USA

**Keywords:** Parkinson’s disease, α-Synuclein, Amyloid, Fibril, Oligomer, Lewy body, Neurodegeneration

## Abstract

The two main pathological hallmarks of Parkinson’s disease are loss of dopamine neurons in the substantia nigra pars compacta and proteinaceous amyloid fibrils composed mostly of α-synuclein, called Lewy pathology. Levodopa to enhance dopaminergic transmission remains one of the most effective treatment for alleviating the motor symptoms of Parkinson’s disease (Olanow, Mov Disord 34:812–815, 2019). In addition, deep brain stimulation (Bronstein et al., Arch Neurol 68:165, 2011) to modulate basal ganglia circuit activity successfully alleviates some motor symptoms. MRI guided focused ultrasound in the subthalamic nucleus is a promising therapeutic strategy as well (Martinez-Fernandez et al., Lancet Neurol 17:54–63, 2018). However, to date, there exists no treatment that stops the progression of this disease. The findings that α-synuclein can be released from neurons and inherited through interconnected neural networks opened the door for discovering novel treatment strategies to prevent the formation and spread of Lewy pathology with the goal of halting PD in its tracks. This hypothesis is based on discoveries that pathologic aggregates of α-synuclein induce the endogenous α-synuclein protein to adopt a similar pathologic conformation, and is thus self-propagating. Phase I clinical trials are currently ongoing to test treatments such as immunotherapy to prevent the neuron to neuron spread of extracellular aggregates. Although tremendous progress has been made in understanding how Lewy pathology forms and spreads throughout the brain, cell intrinsic factors also play a critical role in the formation of pathologic α-synuclein, such as mechanisms that increase endogenous α-synuclein levels, selective expression profiles in distinct neuron subtypes, mutations and altered function of proteins involved in α-synuclein synthesis and degradation, and oxidative stress. Strategies that prevent the formation of pathologic α-synuclein should consider extracellular release and propagation, as well as neuron intrinsic mechanisms.

## Background

Over 10 million people in the world suffer from Parkinson’s disease (PD). The two main pathological hallmarks of PD are loss of dopamine neurons in the substantia nigra pars compacta (SNc), and protein aggregates (called Lewy bodies and Lewy neurites) composed mostly of the protein α-synuclein. Levodopa/carbidopa is currently the most effective treatment for the motor symptoms of PD [1]. Deep brain stimulation also alleviates some motor symptoms and MRI guided focused ultrasound is a developing strategy [2, 3]. However, levodopa does not prevent further neuron loss or Lewy pathology. Over time, patients more frequently experience periods of time in which levodopa does not work and require higher doses of the drug, which increase side effects such as dyskinesias. Furthermore, levodopa does not treat non-motor symptoms including cognitive changes, which are the primary cause for institutionalization, rapid decline, and death. By the time an individual is seen in the clinic for PD symptoms, there is an approximately 90% loss of dopamine neurons in the SNc [4]. Therefore, it is critically important to develop therapeutic strategies to prevent further neuron loss and to prevent the formation of Lewy pathology in other brain areas.

One of the current strategies to prevent PD progression includes targeting the formation, extracellular release and neuron to neuron spread of abnormal α-synuclein aggregates throughout the nervous system. Fibrillar aggregates of α-synuclein corrupt normal, endogenously expressed α-synuclein to form pathologic aggregates which are released and spread throughout the brain. The first line of evidence supporting the release of pathogenic α-synuclein aggregates was the demonstration of aggregates in the cerebrospinal fluid, suggesting it can be released [5]. In addition, studies of PD patients who received fetal nigral grafts in the striatum showed that after several years, the fetal graft tissue harbored Lewy pathology, suggesting the grafted neurons inherited α-synuclein aggregates from neurons from the host brains [6–10]. Staging studies of Parkinson’s disease also support that α-synuclein aggregates appear in the brain in a temporally and spatially predictable manner [11–13]. In early stages, Lewy pathology first appears in the enteric nervous system and the olfactory bulb. In the later stages, Lewy pathology appears in the brainstem, limbic areas, and cortex; appearance in these brain areas coincides with the onset of motor, psychiatric, and cognitive symptoms.

Multiple experimental studies have been published that support that seeds of fibrillar α-synuclein can induce normal α-synuclein to form pathogenic inclusions. Exposure of HEK293 cells stably overexpressing α-synuclein to fibrils generated from recombinant α-synuclein induces the formation of insoluble, phosphorylated, and ubiquitinated aggregates [14]. Exposure of neurons expressing endogenous levels of α-synuclein to fibrils causes formation of α-synuclein inclusions that morphologically and biochemically resemble Lewy neurites and Lewy bodies [15]. Importantly, injection of fibrils into the striatum produces Lewy-like pathology in dopamine neurons of the substantia nigra pars compacta, dopaminergic neuron loss and motor behavior phenotypes similar to PD [16]. α-Synuclein inclusions are also produced in other brain regions relevant for PD, such as the cortex and amygdala. Fibril-induced inclusion formation causes multiple phenotypes, including reduced expression of presynaptic proteins, decreased neuronal connectivity, and reduced spine density [15, 17–19]. Exposure of neurons from α-synuclein knockout mice to α-synuclein fibrils does not produce intracellular inclusions and does not impair neuronal connectivity or synapse formation. Thus, the corruption of endogenous α-synuclein to form insoluble fibrillar aggregates is responsible for neuronal dysfunction. These findings have been replicated by many studies and in other species, such as rats and primates [20–26]. Importantly, fibril exposure of human neuronotypic cells derived from differentiated induced pluripotent cells (iPSCs) induces the formation of α-synuclein inclusions and toxicity [27–30]. These studies demonstrate the potential of human cell models as a platform for screening compounds that potentially mitigate the formation of α-synuclein aggregation and progression of PD. Studies showing that peripheral injection of fibrils induce Parkinson’s disease phenotypes particularly support that aggregated α-synuclein can travel along connected neuron pathways [31–34]. Injection of fibrils into the duodenal and pylori muscularis layers of the gut of wild type mice expressing endogenous α-synuclein results in the formation of Lewy-like pathology to the brainstem and limbic areas that is associated with behavioral defects [32].

Studies of the triggers of the formation of pathological α-synuclein are critical. Endogenous overexpression of α-synuclein, its membrane association with intra- or extracellular transport vesicles, or its cell-to-cell transmission mechanisms can all play critical roles in the formation of α-synuclein fibrils and aggregates. This review article will later explore the most recent findings regarding the multifaceted cell intrinsic contributors to the formation of pathologic α-synuclein inclusions.

There remain several outstanding questions in the field: 1) What conformation of α-synuclein aggregates is responsible for PD phenotypes? 2) Are there selective cell-surface receptors that facilitate α-synuclein uptake? 3) Lewy bodies and Lewy neurites are too large to be released from neurons and taken up by neighboring neurons; are these larger filaments fragmented and released by the neuron and if so, what facilitates fragmentation? 4) Are some neuronal subtypes more susceptible to forming aggregates? 5) α-synuclein is a normal protein in the brain expressed at presynaptic terminals. What initiates the conversion of α-synuclein to β-sheet fibrils? We will discuss these outstanding questions in the following sections.

## Main text

### What is the species of α-synuclein responsible for PD phenotypes?

Many proteins implicated in neurodegenerative diseases convert to a pathologic conformation that induces the endogenous protein to adopt the same conformation and is thus self-propagating [35]. It is critical to define the conformation of α-synuclein that is responsible for this pathogenic templating. Endogenous α-synuclein is primarily expressed in neurons where it is enriched at the presynaptic terminal [36–39]. The normal conformation of α-synuclein is either a disordered monomer or in an alpha-helical, multimeric conformation. α-Synuclein can also convert from the disordered, monomeric form to β-sheets that build by recruiting additional monomer to eventually form protofilaments and amyloid fibrils [40]. Lewy bodies and Lewy neurites in PD brains are characterized by filaments of α-synuclein in the amyloid conformation [41, 42].

Both α-synuclein fibrils and oligomers cause toxicity to a varying extent [43, 44]. The impact of oligomers on the formation of α-synuclein inclusions has historically been difficult because oligomers are inherently transient in nature and rapidly recruit monomeric α-synuclein to form fibrils. The discovery that α-synuclein oligomers can be kinetically trapped provided a major breakthrough, allowing comparisons of the contributions of oligomers and fibrils to PD-phenotypes [43, 45, 46]. Biophysical characterization of the kinetically trapped oligomers shows they are composed of an average of 29 molecules, are approximately 50 nm in length, have anti-parallel β-sheet configuration, contain minimal amyloid conformation, and are not seeding competent, i.e. do not elongate in the presence of monomer. Injection of oligomers into the striatum of mice induces a slight loss of dopamine neurons in the substantia nigra pars compacta (SNc), consistent with previous demonstrations of their toxicity. In comparison, short fibril fragments are approximately 70 nm in length with a parallel β-sheet configuration, amyloid conformation, and are seeding competent. Injection of short fibril fragments into the striatum significantly reduces the number of dopamine neurons in the SNc, reduces dopamine transporter-positive terminals in the striatum, causes seeded formation of α-synuclein inclusions in the SNc, cortex, and amygdala, and produces motor behavior defects [46]. Thus, seeding competent fibril fragments are responsible for PD-related phenotypes. These data are important because while some immunotherapy approaches target fibrillar forms of α-synuclein, other strategies may target monomeric forms and consequently inhibit the function of normal α-synuclein. Because reduction of α-synuclein can cause perturbations in striatal levels of dopamine, which could be an adverse side effect for treating PD [47–49], antibodies that specifically target fibrillar α-synuclein may be most effective in preventing the progression of PD without causing adverse side effects.

Different conformations of fibrils produce distinct phenotypes, suggesting the existence of different “strains” may be responsible for different disease phenotypes [50]. For example, fibrils generated under different buffer and salt conditions can produce either classic fibrils, or flat, twisted assemblies called ribbons [34, 44]. Injection of these different conformations into mice shows that, while the fibrils cause the most robust loss of dopamine neurons and motor behavior defects, ribbons produce α-synuclein inclusions in oligodendrocytes, a characteristic of another α-synucleinopathy, Multiple Systems Atrophy (MSA). A distinct strain of α-synuclein assemblies can also be produced by sequentially passaging the α-synuclein fibrils generated using recombinant α-synuclein [51]. While the classic (non-passaged) fibrils seed formation of inclusions in neurons from endogenous α-synuclein, sequential passaging of fibrils appears to impart a novel conformation such that the passaged fibrils are able to additionally cross-seed tau inclusions similar to those found in Alzheimer’s disease brains. Thus, distinct structural conformations of α-synuclein could generate mixed pathologies by cross-seeding tau. Interestingly, seeds of α-synuclein from MSA brains are more efficient in propagating α-synuclein inclusions when injected into mouse brains than those derived from PD brains [52, 53]. MSA is a much more rapidly progressing α-synucleinopathy compared to PD, suggesting that the strains of seeds in MSA brains are more toxic, forming and spreading more rapidly throughout the brain [50, 54–57]. In particular, seeds of α-synuclein from MSA brains maintain strain characteristics following serial propagation and resist inactivation with formalin, similar to PrP scrapie [55]. Evidence suggests that the MSA strains are conformationally distinct from PD strains [56]. In the future, it will be of great interest to determine how different structural characteristics of these strains could account for different phenotypes of α-synucleinopathies.

### Are there selective cell-surface receptors that facilitate α-synuclein uptake?

**Fig. 1:**
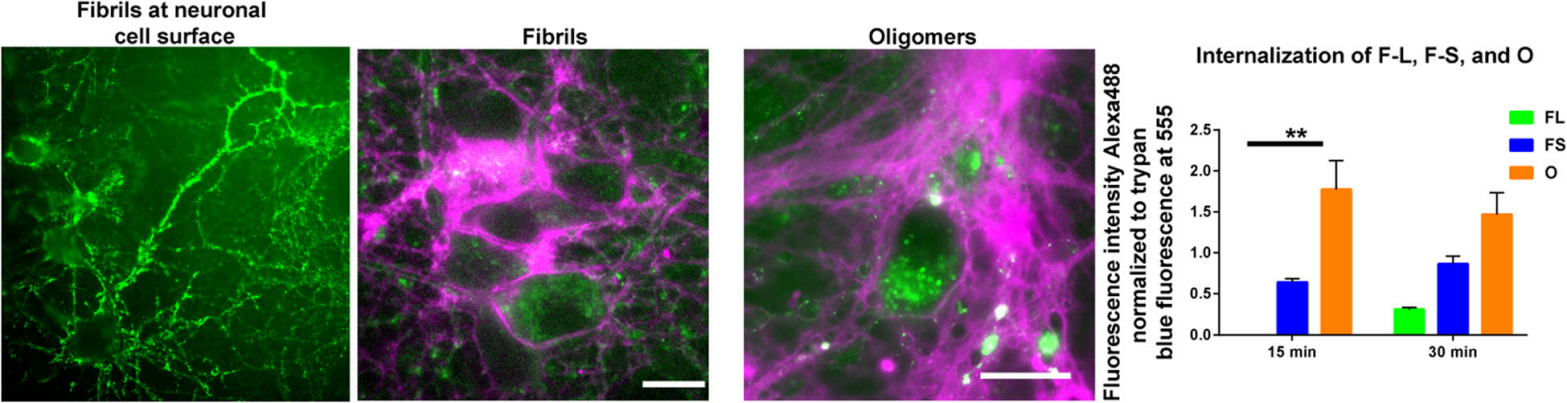
Human α-synuclein fibrils were labeled with Alexa-488. To visualize fibrils at the cell surface, primary hippocampal neurons were incubated at 4 °C for 30 min with Alexa-488 labeled fibrils, rinsed, and images were captured. To visualize internalized Alexa-488 labeled fibrils and oligomers, primary hippocampal neurons were pre-incubated with the fibrils or oligomers for 30 min at 4 °C and then incubated for 15 min at 37 °C. The neurons were rinsed and incubated with trypan blue to quench extracellular Alexa-488 label and to visualize intracellular fibrils and oligomers. When trypan blue binds to proteins on the cell surface, it fluoresces at 560 nm, which is shown in the images as *magenta. The right panel shows quantitation of internalization of unsonicated, long fibrils (F-L), sonicated 50 nm fibrils (F-s) and oligomers (O). From* Froula JM, Castellana-Cruz M, Anabtawi NM, Camino JD, Chen SW, Thrasher DR, Freire J, Yazdi AA, Fleming S, Dobson CM, et al.: Defining alpha-synuclein species responsible for Parkinson’s disease phenotypes in mice. *The Journal of biological chemistry* 2019, 294:10392–10,406

α-Synuclein is an intracellular, cytosolic protein. For the seeded formation of α-synuclein inclusions to occur and propagate to additional brain regions, the fibrils must bind to the neuronal cell surface and gain entry to the cytoplasm. α-Synuclein fibrils bind to the entire cell surface of neurons (Fig. 1). The development of novel fluorescent based techniques allows intracellular fibrils to be distinguished from extracellular fibrils [58] and demonstrates that α-synuclein fibrils are internalized. While α-synuclein fibrils interact with the entire cell surface, fibril uptake and subsequent internalization may depend on specific receptors and/or post-translational modifications on the extracellular domain of transmembrane proteins such as heparin sulfate protein proteoglycans.

Cell surface heparan sulfate proteoglycans (HSPGs) mediate the uptake and internalization of α-synuclein fibrils [59]. HSPGs are glycoproteins with heparin sulfate chains composed of sulfated repeating subunits of N-acetylglucosamine and glucuronic acid. There are 17 distinct types of HSPGs that are found either in the extracellular matrix or on the cell-surface. HSPGs are responsible for internalization of multiple different cargos, such as exosomes, viruses, lipoproteins such as APOE, and amyloid-β [60, 61]. Binding and internalization of α-synuclein fibrils is inhibited by heparin, which competes for binding with cell surface HSPGs, and chlorate, which inhibits sulfation [59]. Interestingly, oligodendrocytes, which have α-synuclein inclusions in MSA, utilize HSPGs for internalization, whereas microglia do not, suggesting that fibril internalization may be cell type selective [62]. To that end, it is possible that targeting HSPGs could prevent the spread of pathologic α-synuclein, as has been previously suggested for tauopathies [63].

Specific cell surface receptors that bind and mediate uptake of α-synuclein fibrils have also been identified. An unbiased proteomic screen showed that α-synuclein fibrils but not oligomers or monomer bind the α3-subunit of the plasma membrane localized Na^+^/K^+^ ATPase [64]. In addition, a screen of a library expressing transmembrane proteins identified that LAG3, amyloid-β precursor-like protein 1, and neurexin1β bind α-synuclein fibrils but not monomeric α-synuclein [23]. Importantly, mice lacking LAG3 show reduced fibril-induced formation of α-synuclein inclusions in the SNc, rescue of fibril-induced dopamine neuron loss, and rescue of motor impairments caused by α-synuclein fibril injections. Interestingly, LAG3 is mainly expressed on activated immune cells such as T-cells and natural killer cells [65]. Activation of the immune system is important for the PD disease process, including activation of microglia and T-cell infiltration into the brain [66–68]. These immune cells may play a role in the propagation of α-synuclein seeds. It will be of great interest to know if LAG3-positive immune cells are involved in the uptake and transmission of α-synuclein aggregates. Recently, neurexin1β, which is enriched at the presynaptic terminal similar to α-synuclein, was shown to mediate uptake of acetylated fibrillar α-synuclein [69], highlighting that post-translational modifications of pathogenic α-synuclein should also be considered when determining selectivity of fibrillar α-synuclein for receptors. It is important to consider, however, that not one specific receptor may be responsible for the uptake of α-synuclein fibrils, implicating the role of a more generalized binding mechanism.

### Are larger filaments fragmented and released by the neuron and if so, what facilitates fragmentation?

Several lines of evidence show that the major disease spreading agents consist of seeding-efficient α-synuclein which are small fibrillar aggregates of around 50 nm in length [46]. Because Lewy bodies and Lewy neurites are too large to account for released aggregates, future studies that examine the mechanisms by which larger filamentous inclusions could fragment within the cell to become new and efficient nuclei for the propagation of α-synuclein inclusions and disease phenotypes will be of great interest. Disaggregation of amyloid fibrils by chaperones produces both monomeric and oligomeric α-synuclein [70]. The chaperone, HSP110, in particular, mitigates formation of α-synuclein aggregates in the brain [71]. These oligomers could be seeding competent oligomers that can be propagated from neuron to neuron. The dissociated monomers could also exist in an inert conformation or a seeding competent conformation that is released by the neuron, as has been demonstrated for tau [72]. It is also possible that lysosomal proteases could cause the fragmentation and release of smaller seeding-competent conformers of α-synuclein. Indeed, low pH increases fibril fragmentation in vitro, a condition that may be replicated in endosomes and lysosomes that have an acidic pH [73]. Because disaggregases and protein degradation pathways are currently being evaluated as putative therapeutic strategies, it is critical to characterize the species of α-synuclein produced by these pathways and whether these treatments reduce or increase the spreading agent.

### Are some neuronal subtypes more susceptible to forming aggregates?

**Fig. 2:**
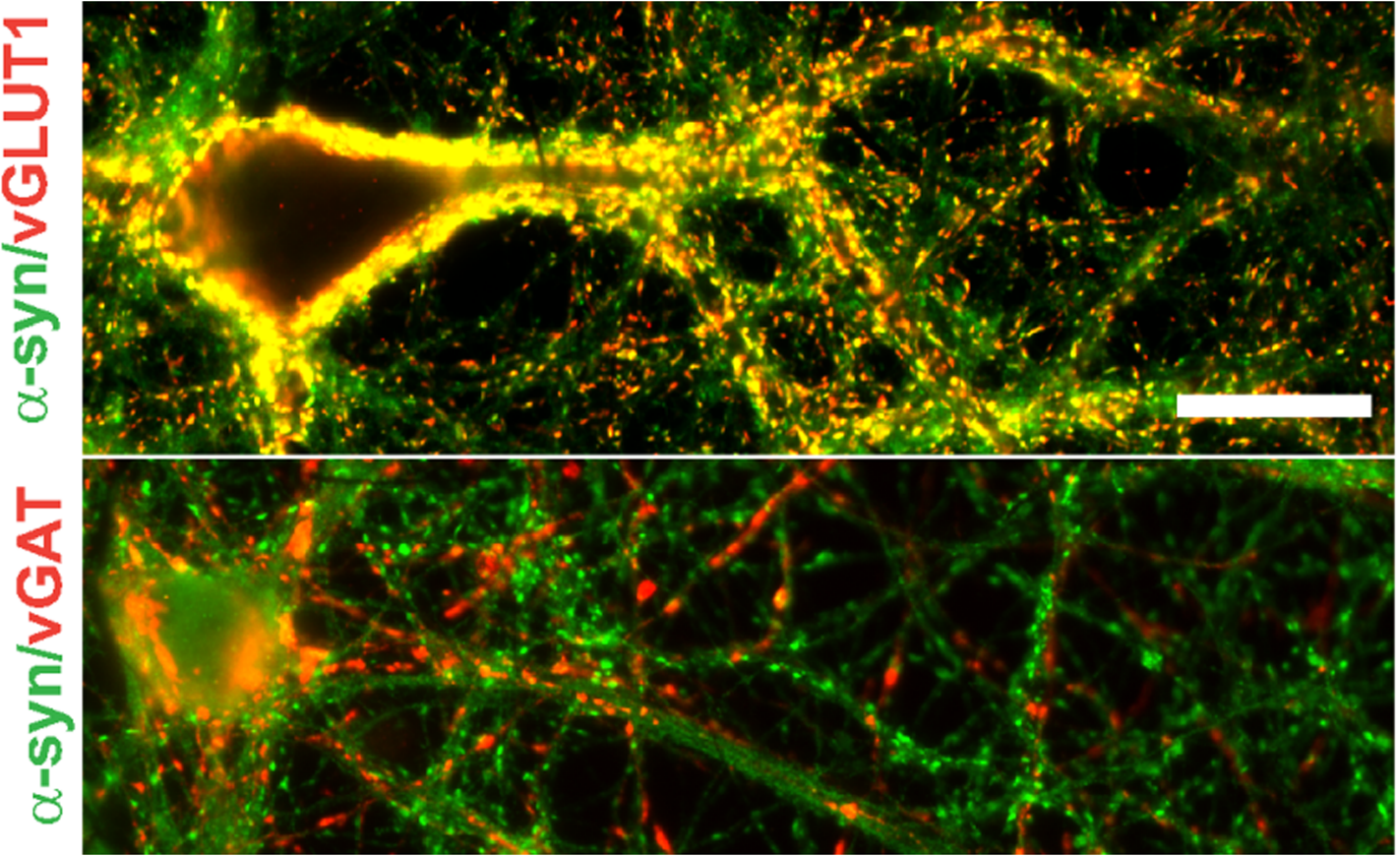
Immunofluorescence was performed in primary hippocampal neurons using antibodies to α-synuclein and either vGLUT1 to identify glutamatergic presynaptic terminals or vGAT to identify GABAergic presynaptic terminals. Colocalization of α-synuclein with vGLUT1 is visualized as yellow in the merged image. Image from: Froula JM, Henderson BW, Gonzalez JC, Vaden JH, McLean JW, Wu Y, et al. alpha-Synuclein fibril-induced paradoxical structural and functional defects in hippocampal neurons. *Acta Neuropathol Commun.* 2018;6(1):35

In α-synucleinopathy brains, some neuronal subtypes show abundant Lewy pathology while it is absent in other neuronal subtypes. For example, dopaminergic, noradrenergic, cholinergic and glutamatergic neurons show abundant α-synuclein aggregates while most GABAergic neurons are mostly spared [41, 74–79]. Levels of α-synuclein expression may also determine susceptibility to forming inclusions [76, 78, 80], analogous to in vitro experiments in which increasing concentrations of α-synuclein enhances the kinetics of fibrillization. This could explain the finding that α-synuclein is enriched at the presynaptic terminal of glutamatergic neurons but not GABAergic neurons, making them more susceptible to Lewy pathology [81] (Fig. 2). However, neurotransmitter phenotype is not the sole determinant of susceptibility. For example, dopaminergic neurons in the SNc are more vulnerable than neighboring dopamine neurons in the ventral tegmental area (VTA). However, it should be noted that few studies have performed stereological quantification of neurons in the VTA and the one study to do so reported a loss of neurons in this dopaminergic regions [82, 83]. Further, glutamatergic neurons in the Cornu Ammonis (CA) that express the transcription factor Math2 have higher levels of α-synuclein and form more abundant inclusions when compared to low Math2 expressing neurons in the dentate gyrus [84].

It is also possible that some neuronal subtypes express lower levels of components of degradative machinery which would lead to higher levels of α-synuclein. For example, glutamatergic neurons express lower levels of BAG3, which is involved in autophagy. Reducing BAG3 increases formation of tau aggregates, whereas increasing BAG3 reduces tau aggregates [85]. BAG3 expression may similarly affect α-synuclein levels by potentially facilitating α-synuclein degradation [86]. Finally, inclusions in MSA appear in neurons and oligodendrocytes called glial cytoplasmic inclusions (GCI) [87, 88]. Because PDGFRA+ and O4+ oligodendrocyte progenitors express higher levels of α-synuclein than mature oligodendrocytes, it is possible that impaired oligodendrocyte maturation may lead to the accumulation of α-synuclein, making these cells more susceptible to α-synuclein aggregation [89]. Interestingly, a small population of PD patients with a G51D-α-synuclein mutation also shows α-synuclein aggregates in oligodendrocytes, as well as in neurons [88]. Future studies investigating factors that make oligodendrocytes more susceptible to forming Lewy pathology will further our understanding of the mechanisms of MSA and other α-synucleinopathies. With emerging studies utilizing single cell RNA sequencing to identify distinct subtypes of neurons, researchers in the future will be better able to characterize the factors contributing to selective vulnerability.

### What causes the conversion of α-synuclein from a normal conformation to a β-sheet, seeding competent conformation?

#### Total α-synuclein levels

Exposure of neurons to fibrils generated from recombinant α-synuclein induces endogenously expressed α-synuclein to form inclusions that closely resemble those found in PD brains, allowing researchers to model inclusion formation, associated impact on neuronal function and research targets that could prevent aggregation [15]. However, what causes normal α-synuclein to convert to a pathogenic form in humans [90]? α-Synuclein normally exists in a disordered conformation or an α-helical, membrane associated, multimeric conformation. It is critical to understand what makes α-synuclein convert to forming β-sheets that stack to form protofibrils and eventually fibrils [90]. In a test tube, increased concentration of α-synuclein enhances kinetics of fibrillization [91]. The cytosol of the neuron is likely similar to test tube conditions in that higher levels of α-synuclein increase the susceptibility of neurons to form inclusions. The hypothesis that levels of α-synuclein contributing to its propensity to fibrillize is supported by multiple genomic studies showing that duplication or triplication of the α-synuclein gene, SNCA, causes PD [92–94]. Even polymorphisms in the distal enhancer region of SNCA increase risk for PD and cause a slight (1.06 time) increase in α-synuclein [95]. Mutations in other genes linked to PD may also impact α-synuclein expression levels. For example, dominant inherited mutations in leucine-rich repeat kinase 2 (LRRK2) cause PD [96]. Additionally, neurons from BAC transgenic mice expressing the G2019S-LRRK2 mutations have higher total levels of cytosolic α-synuclein which could account for the increased fibril-induced α-synuclein aggregates found in mutant LRRK2 expressing mice [97]. Multiple functional roles have been assigned to LRRK2, but several studies point to a role for LRRK2 in autophagosome/lysosome degradation pathways [98, 99]. PD-associated mutations in LRRK2 increase mRNA translation, so it is possible that mutant LRRK2 increases α-synuclein levels by increasing synthesis and preventing degradation [100]. In addition, mutations in GBA, which encodes for glucocerebrosidase (GCase), are one of the most common risk factors for PD [101]. GCase is a lysosomal enzyme that hydrolyzes glycosphingolipids. Mutations in GCase reduce its enzymatic function, causing a buildup of these lipids and consequently impairing lysosomal function [102]. Mutations in GBA also cause an accumulation of total α-synuclein and consequently, α-synuclein aggregates [103]. In addition to gene mutations, genome wide association studies show that sequence variations in genes associated with degradation pathways associate with PD, including variations of CHMP2B, TMEM175, SCARB3 and BAG3 [104, 105]. Since PD mostly manifests in individuals over 65 years of age, it is possible that even slight impairments in protein degradation pathways could lead to a buildup of α-synuclein over decades.

Levels of α-synuclein at specific subcellular compartments may also play an important role in its susceptibility to form aggregates. Lewy neurites in axons form before Lewy bodies in the soma [106, 107]. Addition of fibrils to neurons that endogenously express α-synuclein induces formation of α-synuclein inclusions first in axons [15]. The enrichment of α-synuclein at the presynaptic terminal is the mostly likely reason for initiation of aggregate formation in the axon. Interaction of α-synuclein with partners such as VAMP2, CSPα, and synapsin may also enhance presynaptic enrichment of α-synuclein [108–111].

#### Impaired mitochondrial function

It has been known for decades that impaired mitochondrial function causes abnormal α-synuclein aggregation. Chronic administration of 1-methyl-4-phenyl-1,2,3,6-tetrhydropyridine (MPTP) to mice and primates closely recapitulates symptoms of PD and causes loss of neurons in brain regions relevant for PD: dopamine neurons in the SNc, noradrenergic neurons in the Locus Coeruleus, serotonerigic neurons in the Raphe Nucleus, and cholinergic neurons in the Pedunculopontine brainstem nucleus [112]. MPTP is taken up by neurons and metabolized to MPP+ which inhibits mitochondrial complex I. Chronic, but not acute, administration of MPTP leads to formation of α-synuclein aggregates [112, 113]. Interestingly, chronic administration of MPTP to α-synuclein knockout mice prevented behavioral defects and MPTP-induced loss of dopamine neurons, suggesting that complex I mediated parkinsonism phenotypes depend on the corruption of α-synuclein [113]. The pesticide rotenone is another complex I inhibitor; chronic administration of rotenone induces formation of α-synuclein aggregates [114, 115]. Inhibition of complex I causes increased oxidative stress, resulting in the oxidation and nitration of α-synuclein which increases its propensity to aggregate [116]. More research studying the contribution of pesticides and environmental toxins to α-synuclein aggregation will pave new therapeutic avenues for the prevention of Lewy pathology [117].

#### Association with membranes

Normally in neurons, the N-terminal domain of α-synuclein adopts an amphipathic α-helical conformation that associates with membranes where it assembles into multimers [118–122]. Disruption of this α-synuclein membrane association increases its propensity to form pathological aggregates [120]. Mutations in the N-terminal domain that prevent α-synuclein membrane binding increase aggregate formation in vitro*,* and when expressed in cells [120]. Reductions in the ratio of membrane lipids to total α-synuclein causes an excess of monomeric α-synuclein and enhances amyloid aggregation [123]. Biophysical studies demonstrate that the role of membranes in aggregation is not as simple as membrane bound or cytosolic, however. Lipids and membrane vesicles increase the kinetics of α-synuclein amyloid formation. The central hydrophobic NAC domain (amino acids 61–95) facilitates α-synuclein amyloid aggregate formation [124]. When α-synuclein is membrane bound, the NAC domain can toggle between being buried in the membrane or exposed to the cytosol [125]. Upon exposure to the cytosol, the NAC domain recruits additional α-synuclein monomer and initiates the formation of aggregates that then dissociate from the membrane [126]. Mutations in α-synuclein (A30P and G51D) that cause Parkinson’s disease increase exposure of the NAC domain to the cytosol and away from the membrane, which could be the initiating factor in the formation of Lewy pathology in patients with these mutations [127]. Lipid composition also plays an important role in α-synuclein association and aggregation [126]. α-Synuclein preferentially associates with anionic lipids typically found in synaptic vesicles, such as 1,2-Dioleoyl-sn-glycero-3-phosphoethanolamine (DOPE), 1,2-Dioleoyl-sn-glycero-3-phosphocholine (DOPC), 1,2-Dioleoyl-sn-glycero-3-phosphoserine (DOPS), 1-palmitoyl-2-oleoyl-sn-glycero-3-phospho-l-serine (POPS), and cholesterol [128]. Exposure of the NAC domain and amyloid aggregation is enhanced in the presence of short chain fatty acids, oleic acid (30,527,540 and 30,517,862), or 1-palmitoyl-2-oleoyl-sn-glycero-3-phospho-l-serine (POPS) [126, 128–130]. Oxidative modification to lipids caused by reactive oxygen species generated from damaged mitochondria could also enhance exposure of the NAC domain to the cytosol. Therefore, compounds that enhance membrane association of the NAC domain could serve as potential disease modifying interventions by preventing α-synuclein aggregation and subsequent spread throughout the brain [131, 132].

Small lipid vesicles called exosomes may play a role in the spread and toxicity of α-synuclein. Exosomes are released by neurons and other cell types and can be formed either by direct budding from the plasma membrane, or are released internal vesicles of multivesicular bodies. α-Synuclein can be released from the cell in association with exosomes. Some studies have suggested that exosomal α-synuclein might play a role in enhancing the spread of aggregates throughout multiple brain regions and form neuronal toxicity [133–136]. A recent study demonstrated that overexpression of the chaperone, 14–3-3, prevented neuron to neuron propagation of α-synuclein but paradoxically increased release of non-toxic exosomal α-synuclein [137]. Thus, the role of exosomal α-synuclein in the propagation of pathology requires further research. Fibrillar α-synuclein associated with exosomes can be detected in fluids such as cerebral spinal fluid and may provide an important biomarker for diagnosis and early detection of PD [138, 139].

## Conclusions

The findings that fibrillar seeds of α-synuclein can template endogenous α-synuclein to form polymers which ultimately spread throughout the neuron and the nervous system made a substantial contribution to the PD field. It is still important to not lose sight of whether or not the spread of α-synuclein is a primary driver of PD progression [90]. There exist multiple neuron-intrinsic mechanisms that likely contribute to conversion of normal α-synuclein to abnormal aggregates. This is especially relevant when examining cases of mutation-driven versus idiopathic PD. For instance, is PD progression in these patients solely a consequence of a genetic initiator or trigger of fibril formation (e.g. SNCA polymorphisms that result in α-synuclein overexpression), notwithstanding the subsequent propagation of α-synuclein? That is to say, if an individual’s genetic profile hinders or enhances the propensity of α-synuclein aggregates to form in mutant-driven cases of PD, does the manner in which α-synuclein is spread still hold clinical relevance? In addition, it is worth noting that 90% of PD cases are not driven by a known mutation. For idiopathic cases, questions like these remain unclear. Therefore, whether it is the templated seeding of α-synuclein presently discussed or some epiphenomenal cause that may not be directly related to the consequential dissemination of pathologic α-synuclein, further studies investigating external factors that might initiate α-synuclein aggregation to disease phenotypes remain crucial.

Still, research on the extracellular release of α-synuclein and its inheritance by connected neurons opens up the field for novel treatments that can potentially halt PD in its tracks, including immunotherapy to prevent the neuron to neuron spread of aggregated α-synuclein. Recently, administration of monoclonal antibodies for α-synuclein, PRX002 and BIIB054 were demonstrated to have a favorable safety, tolerability and pharmacokinetic profile [140–142]. Affitope PD01A which triggers an immune response to α-synuclein also has a favorable safety profile. Hopefully, it will soon be determined that immunotherapy to α-synuclein can slow the progression of PD.

Prodromal detection of α-synuclein aggregates is also critical when developing therapies that target α-synuclein aggregation. Older individuals with minimal motor symptoms show loss of dopamine terminals in the striatum, loss of dopamine neurons, and robust α-synuclein inclusions [143]. These data suggest that prodromal individuals can be identified whereby targeting α-synuclein could most effectively prevent development of more severe motor symptoms. Hopefully, more sensitive techniques such as RT-QuIC to identify fibrillar α-synuclein in biofluids [139], or imaging methods to detect the early loss of dopamine terminals in the striatum [4], will help with early diagnosis before the almost complete loss of dopamine neurons.

To discover a novel therapeutic that prevents abnormal α-synuclein requires a comprehensive understanding of the initial triggers of α-synuclein aggregation and how aggregation propagates within neurons and across the brain. To this end, there remain several outstanding questions and challenges for the field:

1. Precisely defining, with rigorous biophysical methods, the pathogenic conformation of α-synuclein.
2. Determining whether specific receptors facilitate uptake of fibrillar α-synuclein or if post-translational modifications to extracellular domains of transmembrane proteins facilitate fibril binding and uptake.
3. Understanding how small species of fibrillar aggregates are generated and released by the neuron.
4. Discovering the cell intrinsic mechanisms that trigger α-synuclein aggregation. For example, increased expression or decreased degradation of α-synuclein can initiate the formation of small amyloid aggregates that, over the course of a lifetime, can assemble to form Lewy bodies and Lewy neurites. Impairments in mitochondrial function or an increase in oxidative stress can induce protein modifications that facilitate the conversion of α-synuclein to a fibrillar pathway. Changes in membrane composition or changes in expression of binding partners can initiate α-synuclein aggregation.
5. Determining if some neuronal subtypes are more susceptible to forming aggregates due to reduced levels of protein degradation pathways, reduced lipid metabolizing enzymes, or higher levels of oxidant stress, as examples.
6. Determining if and how early formation of aggregates impairs neuronal function. By the time the aggregates assemble into Lewy bodies and the neurons die, possible interventions are likely too late.

Discovering compounds that can prevent the nucleation and accumulation of α-synuclein fibrillar species will hopefully lead to the first therapeutics that halt PD in its tracks.

## Data Availability

Data sharing is not applicable to this article as no datasets were generated or analyzed during the current study. All data was published previously.

## Abbreviations

HSPGs: Heparan sulfate proteoglycans
MPTP: 1-methyl-4-phenyl-1,2,3,6-tetrhydropyridine
MSA: Multiple Systems Atrophy
PD: Parkinson’s disease
SNc: Substantia nigra pars compacta
